# Linkage-based genome assembly improvement of oil palm (*Elaeis guineensis*)

**DOI:** 10.1038/s41598-019-42989-y

**Published:** 2019-04-29

**Authors:** Ai-Ling Ong, Chee-Keng Teh, Qi-Bin Kwong, Praveena Tangaya, David Ross Appleton, Festo Massawe, Sean Mayes

**Affiliations:** 1 Biotechnology & Breeding Department, Sime Darby Plantation R&D Centre, Serdang, Selangor Malaysia; 2 School of Biosciences, University of Nottingham Malaysia, Semenyih, Selangor Malaysia; 3 School of Biosciences, University of Nottingham, Leicestershire, UK

**Keywords:** Agricultural genetics, Plant breeding

## Abstract

Meiotic crossovers in outbred species, such as oil palm (*Elaeis guineensis* Jacq., 2n = 32) contribute to allelic re-assortment in the genome. Such genetic variation is usually exploited in breeding to combine positive alleles for trait superiority. A good quality reference genome is essential for identifying the genetic factors underlying traits of interest through linkage or association studies. At the moment, an AVROS *pisifera* genome is publicly available for oil palm. Distribution and frequency of crossovers throughout chromosomes in different origins of oil palm are still unclear. Hence, an ultrahigh-density genomic linkage map of a commercial Deli *dura* x AVROS *pisifera* family was constructed using the OP200K SNP array, to evaluate the genetic alignment with the genome assembly. A total of 27,890 linked SNP markers generated a total map length of 1,151.7 cM and an average mapping interval of 0.04 cM. Nineteen linkage groups represented 16 pseudo-chromosomes of oil palm, with 61.7% of the mapped SNPs present in the published genome. Meanwhile, the physical map was also successfully extended from 658 Mb to 969 Mb by assigning unplaced scaffolds to the pseudo-chromosomes. A genic linkage map with major representation of sugar and lipid biosynthesis pathways was subsequently built for future studies on oil related quantitative trait loci (QTL). This study improves the current physical genome of the commercial oil palm, and provides important insights into its recombination landscape, eventually unlocking the full potential genome sequence-enabled biology for oil palm.

## Introduction

Oil palm (*Elaeis guineensis Jacq*.) is the only major crop member in the *Arecaceae* family producing edible oil^1^. Being an efficient oil crop, the average oil yield of oil palm is 4 metric tons per hectare every year, which is approximately 10 times higher than soy (*Glycine max*.)^2,3^. Palm oil is currently the most traded vegetable oil, supplying one third of global vegetative oil^3,4^ on less than 6% of the world’s agricultural land dedicated to oil crops. Despite yield superiority, most of the commercially grown oil palm materials have undergone only a limited number of generations of selection since 1920’s due to long selection cycles (12–19 years). In recent decades, the oil palm industry has put major effort into genomics and DNA marker research, primarily to hasten breeding progress. To achieve this, marker-assisted selection (MAS) has been introduced into breeding programmes to allow estimation of the breeding values in palms as early as the nursery stage^5,6^.

Marker-assisted selection usually requires prior knowledge on the distribution of quantitative trait loci (QTL) for a targeted trait in the genome. Over 20 years, linkage maps from various backgrounds of oil palm have been constructed. The first restriction fragment length polymorphism (RFLP)-based linkage map for oil palm was reported in a self-pollinated palm from the *Binga* origin in Africa^7^. Similar efforts were then continued mainly using microsatellites for higher throughput and reproducibility, followed by QTL localization onto these maps^8–10^. The family-based mapping approach was found to be useful for detecting major QTLs, however QTLs were not consistent across populations (with a few exceptions). Consensus maps of one or a number of progenies, using single nucleotide polymorphism (SNP) markers have been constructed to maximize marker density^11,12^.

Genetic maps reflect the actual inheritance of loci into their offspring based on the patterns of recombination during meiosis. Recombination is generally modelled to be a Poisson distribution, with individual chromosomes in each individual gamete expected to undergo at least one recombination event (as this is needed for correct disjunction), but generally no more than three. These are then converted to centiMorgans (cM) through the use of mapping functions, such as the Haldane or Kosambi^13–15^. This limitation in the extent of recombination in gametes also restricts the rate at which alleles close together on the same chromosome are separated in the offspring. Recombination is also not randomly distributed across the genome, with larger genome species often having high levels of recombination at the ends of the chromosome, but little recombination in the heterochromatic and centromeric regions of the chromosome, despite genes still being present (e.g. in wheat^16,17^). Recombination within an oil palm progeny limits the potential combination of alleles that can be achieved by breeding and, particularly, the number of offspring that need to be generated to be able to identify those with the desired combinations.

Conventional oil palm breeding is often based on using test-crosses to evaluate the breeding potential of a parent. To assess parents and develop General Combining Ability (GCA; additive effects) and Specific Combining Ability (SCA; non-additive effects) for parental selection, the number of crosses assessed is more important than the absolute number of test-cross palms recorded (Corley and Tinker, 2015). In practice, 16 to 96 palms per cross in a trial are planted. This limited number of individuals from a single cross has focused attention on genome-wide association studies (GWAS). Teh and colleagues evaluated two multi-parent populations (2,045 *tenera* palms) for mesocarp oil yield^18^ using a high-density OP200K SNP array^19^. The GWAS was based on the published physical genome of a *pisifera* palm of *E*. *guineensis*^20^. Only 60% (55,054) of the SNPs informative in the cross were mapped onto the reference genome. Furthermore, the size of the pseudo-molecule physical map is merely one third (657.96 Mb) of the full expected 1.8-Gb genome size and even the total scaffold lengths only added up to 1.53 Gb, which is still incomplete. Hence, it is critical to further improve the reference genome to unlock the full potential of genome-enabled biology for oil palm. Linkage maps can contribute to the placement of scaffolds into the pseudo-chromosomes. The maps also give important information on the relative recombination distance between loci from different pools of germplasm. In combination with a physical genome sequence, they can indicate the presence of inversions, translocations and duplications which may influence how effectively important gene combinations can be brought together through crossing and marker selection.

For the oil palm industry, the commercial planting materials are dominated by high-yielding *tenera* palms, which are derived from crosses between Deli *dura* and AVROS *pisifera*. The paternal *pisifera* is more heterozygous due to lower levels of inbreeding (although potentially from an extremely narrow genetic base) while the maternal *dura* is normally the descendent from generations of self-pollination or sib-mating from a limited gene pool^21^. Genetic differences, such as large scale structure variations among the genomes of *dura*, *tenera* and *pisifera* can confound QTL results, predicted genetic interactions and breeding objectives, resulting in inconsistencies of QTL positions between different origins. Currently, the extent of such variation in oil palm is still unclear.

In this study, we aimed (1) to construct an ultrahigh-density reference linkage map for genome sequence improvement using the largest commercial *tenera* population reported to date; (2) to integrate linkage maps of both parents and their progeny to the physical genome; (3) to map a genic SNP subset representing genes in important biochemical pathways for future QTL analyses, especially oil yield traits.

## Materials and Methods

### Mapping population and DNA preparation

A total of 295 F_1_ *tenera* palms derived from a maternal Deli *dura* and paternal AVROS *pisifera* (278 x TT41/4) family were selected as a mapping population. The same origin (Deli *dura* x AVROS *pisifera*) was represented in the pool of 59 accessions used for array design. Fresh tissue was sampled from the third leaflet of each seedling. The population is currently at juvenile stage and maintained at Sime Darby Plantation, Malaysia. Total genomic DNA was isolated from 0.1 g of the leaf tissue using the DNAeasy Plant Mini Kit (Qiagen, Germany). The DNA quality was then quantified on a 0.8% agarose gel using known standards.

### SNP identification and genotyping

The mapping population (genomic DNA = 25 ng/μl) was genotyped using the 200,000 SNPs accommodated on the published OP200K Infinium array (Illumina, USA) on the Infinium iScan platform according to the manufacturer’s recommendations^19^. The raw intensity SNP data were analysed with GenomeStudio version 2001.1 with genotyping module version 1.8.4. Using a GenCall score cut-off of 0.15, auto-clustering of the SNPs was done. The SNP clustering was done and was manually confirmed by visual inspection. The SNP call were exported into the PLINK program for minor allelic frequency (MAF) and call-rate filtering^22^.

### Mining of genic SNP markers and annotations

All oil palm transcriptome sequences available in GenBank^23^ were downloaded and combined with an in-house developed transcriptome database^24,25^. The transcriptome dataset was then aligned to the published oil palm genome^20^ using BLAT^26^ to identify gene structures. The SNP markers which were found in the published genome, residing within an interval between the first exon and the last exon of the gene structure were marked as ‘genic’. This step was done using an in-house Perl script. The SNP-tagged transcripts were annotated by referring to KEGG^27^ and UniProt^28^ databases using BLASTN^29^ sequence homology search. This allowed us not only to identify SNPs within genes with a potential influence on important traits, but also to construct a reference genic SNP-based linkage map in oil palm.

### Construction of genomic, genic SNP-based linkage maps and integration with physical map

Polymorphic SNPs were identified where at least one of the parents was heterozygous. Subsequently, polymorphic SNPs were discarded if call rate <95% or if they had high segregation distortion using False Discovery Rate (FDR) corrected method with cutoff at p-value > 0.05. Next, linkage maps were constructed using Lep-MAP3^30,31^ for large SNP datasets, which was especially developed for outbred families like oil palm with an underlying maximum likelihood (ML) method. *Filtering modules* was used for marker quality checking, followed by running the *Separate Chromosomes* module for binning the assayed markers into linkage groups with optimised LOD values ranging from 4 to 30. The *Join Singles* module assigned singular markers to existing linkage groups to maximize the map-abilities of the total input marker group. Lastly, the *Order Markers* module ordered the binned markers according to the Kosambi mapping function for conversion of recombination frequencies into map distances (in unit centiMorgan, cM). The option “sexAveraged = 1” was selected along with *Order Markers* to join the maps of both parents. The polymorphic genic SNP subset was subsequently loaded into JoinMap 5^32^ for linkage mapping to compare the performance of different mapping tools as well as to validate the mapping results. The parameters were set to default, the mapping function used here was ML for estimations of marker distances (in unit cM). All linkage maps were visualised using an R script with minor modifications^33^ and Mapchart^34^.

Common SNP markers were used to carry out a comparative analysis between the linkage maps in cM distance (*dura* parent, *pisifera* parent and *tenera* progeny) and the physical map in Mb distance, with each chromosome visualised in plots. The scatter plots to visualise the integration of the genomic SNP-based genetic map and the physical map was plotted using an in-house R script. Each chromosome was plotted separately to visualise the comparisons between both parental and progeny (combined) maps against the genome assembly.

### Improvement of genome coverage

SNP markers from OP200K were mapped against the published oil palm assembled sequences, available at NCBI under BioProject accessions https://www.ncbi.nlm.nih.gov/bioproject/PRJNA434010 and the assembly at https://www.ncbi.nlm.nih.gov/assembly/GCA_000442705, for the whole genome shotgun sequencing project with all scaffolds including the unplaced scaffolds on the physical map. The progeny (combined) linkage map was applied to bin and anchor the unplaced scaffolds to the physical map. The improvement of genome coverage was quantified based on the sum of the newly anchored scaffolds and their total length in unit of Mb for each of the pseudo-chromosomes.

## Results

### Identification of genomic and genic SNPs

A total of 34,315 genomic SNPs was identified to be polymorphic in the mapping population, and the number was reduced to 29,057 SNPs after removing the segregation-distorted markers or those with call rate <95%. For linkage analysis, 27,890 SNPs and the subset of 2,314 genic markers were mapped, respectively (Table 1).

**Table 1:** A summary of the Deli *dura* x AVROS *pisifera* linkage map using genomic SNP markers, the selected genic SNP markers and estimated recombination rate.

Linkage group	Linkage Length (cM)	No. of linked SNPs	No. of SNPs mapping in published genome	Chromosome size (Mb)	No. of selected genic SNPs	Average Recombination rate (cM/Mb)
1	122.06	2422	1803 (74.4%)	68.44	215	1.8
2	119.96	3533	1535 (43.4%)	65.56	194	2.0
2.2	9.69	248	199 (80.2%)			
3	103.24	2582	1959 (75.9%)	60.06	260	1.7
4	121.09	2235	1203 (53.8%)	57.25	134	2.1
5	33.04	1281	659 (51.4%)	51.96	84	0.8
5.2	7.83	124	75 (60.5%)		18	
6	81.23	1752	1079 (61.6%)	44.36	117	1.8
7	25.67	889	758 (85.3%)	43.46	120	0.6
7.2	12.63	217	198 (91.2%)			
8	89.42	2512	1312 (52.2%)	40.20	178	2.2
9	61.39	1723	889 (51.6%)	38.06	120	1.6
10	74.41	2912	1675 (57.5%)	31.90	310	2.3
11	50.81	1012	783 (77.4%)	30.07	98	1.7
12	84.92	1312	754 (57.5%)	28.80	123	2.9
13	27.37	620	423 (68.2%)	27.81	41	1.0
14	56.76	1553	1037 (66.8%)	24.38	165	2.3
15	42.48	586	541 (92.3%)	24.31	88	1.7
16	27.70	377	328 (87.0%)	21.37	49	1.3
Total	**1151**.**70**	**27890**	**17210** (**61**.**7%**)	**657**.**99**	**2314**	
Mean	**60**.**62**					**1**.**8**

cM – centiMorgan, the percentage of 200,000 SNP markers mapped in the reference genome in parentheses.

*The linkage group number follows chromosome number of the published genome^20^.

### Construction of genomic and genic SNP-based linkage maps

**Figure 1:**
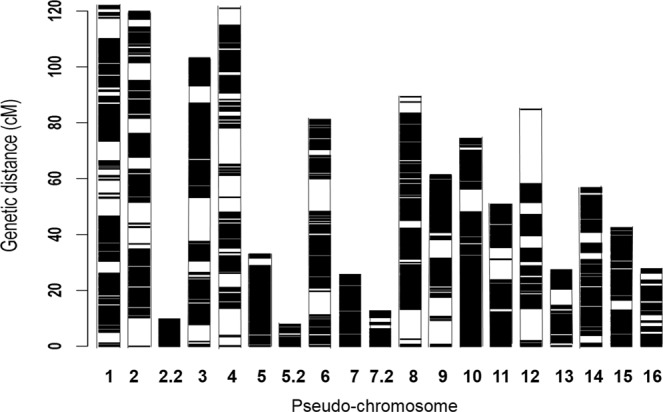
An ultrahigh-density Deli *dura* x AVROS *pisifera* linkage map with 27,890 SNP markers. The left scale represents the linkage length in unit centiMorgan (cM), whereas the number below each bar represents the linkage group number/pseudo-chromosome.

Two linkage maps of Deli *dura* x AVROS *pisifera* were constructed using different set of SNPs. The first set consisted of 27,890 genomic SNPs that were successfully assigned to the 19 linkage groups (LGs; representing the 16 chromosomes of oil palm), spanning 1,151.7 cM with an average mapping interval of 0.04 cM (Table 1). Out of 27,890 genomic SNPs, 17,210 SNPs (61.7%) were found in the published genome. The LG number presented in Fig. 1 is ordered by chromosome number for better traceability. The oil palm consists of a diploid genome with 16 chromosome pairs. In the linkage map, we observed three chromosomes (2, 5 and 7) fragmented into two LGs, respectively. By excluding the three fragments, the longest LG 1 (122.06 cM) and the shortest LG 13 (27.37 cM) which also reflected the similar ranking in terms of their chromosome sizes. On average, 60.62 cM per LG was observed. The ultrahigh-density linkage map is given in Fig. 1. The largest interval between markers was 26.68 cM and located on LG 12.

**Figure 2:**
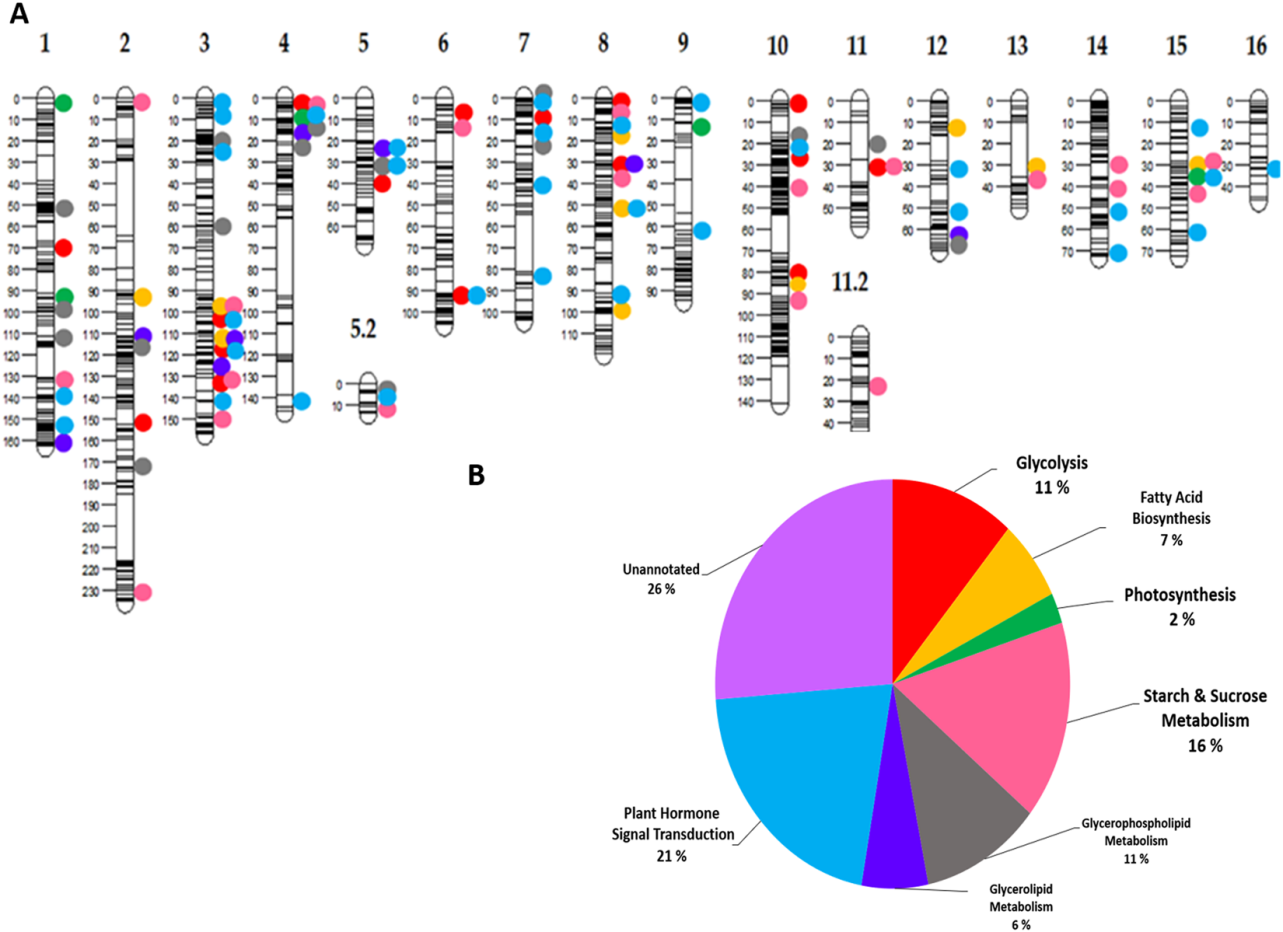
(**A**) A high-density genic SNP-based linkage map of the Deli *dura* x AVROS *pisifera* family with 2,314 SNP markers, (**B**) A pie chart depicting the distribution of the top-ranked pathway membership for the annotated genic SNP markers.

A set of 2,314 genic markers were selected from the total 27,890 linked SNPs to construct another map with lower complexity, based on genic SNP markers alone, designed for future QTL analyses. The map spanned 1,733 cM with an average mapping interval of 0.75 cM (Supplementary Table 1). The obtained 18 LGs was still higher than the expected 16 LGs for the oil palm genome (Fig. 2A). Two LGs (5 and 11) were found to be fragmented in this map. In Fig. 2B, the annotation results indicate that the genic markers were mainly represented by seven pathways based the annotated genes/pathways, including (1) plant hormone signal transduction (25%), (2) starch and sucrose metabolism (19%), (3) glycolysis (13%), (4) glycerolphospholipid metabolism (13%), (5) fatty acid biosynthesis (8%), (6) glycerolipid metabolism (7%), (7) photosynthesis (3%). The pathway annotations were superimposed on the linkage map (Fig. 2A). The genomic SNPs and the subset of genic markers were used to construct maps using Lep-MAP3 and JoinMap 5, respectively. The common SNP markers were compared, showing high concordance of marker ordering (Supplementary Fig. 1).

### The integration between the linkage maps and the physical map

**Figure 3:**
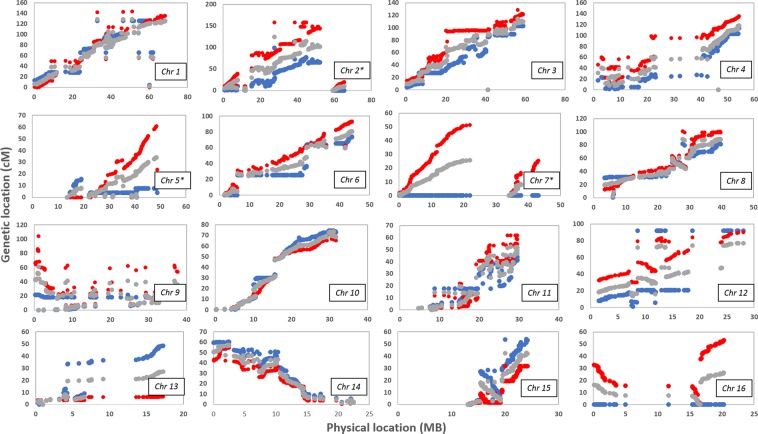
Scatter plots of integration between genetic linkage order and physical marker order throughout the oil palm genome. Blue dots represent the *dura* genetic map and red dots represent the *pisifera* genetic map. Grey dot represents the *tenera* combined map. (*Fragmented linkage groups with shorter length, including LG 2.2, LG 5.2 and LG 7.2 were assigned to Chromosome 2, Chromosome 5 and Chromosome 7, respectively).

The genomic linkage distances of Deli *dura* (Parent 1), AVROS *pisifera* (Parent 2), and their *tenera* progeny were compared to the physical distances. The comparison results across 16 chromosomes are illustrated in Fig. 3. In general, the common SNPs among *dura*, *pisifera* and *tenera* shared the same linkage order due to parallel marker distributions against their physical positions. Lower recombination rates were observed on nine chromosomes for the *dura* parent, compared to the *pisifera* especially on Chromosome 7 and Chromosome 16. Higher rates of recombination for *dura* were only observed on Chromosomes 13 and 15. The combination of the *dura* and *pisifera* linkages map is through combining pairwise distances, so the recombination rate of the *tenera* is intermediate to the parental values. Interestingly, recombination on Chromosomes 7 and 16 of the *tenera* was due to one-sided inheritance of genetic polymorphism from the *pisifera* parent, alone. The order obtained between genetic and physical maps were generally good, except for Chromosomes 9, 11 and 12. Some evidence is seen for potential inversions of chromosome segments between the linkage population and the reference genome, such as on Chromosome 15 (15–20 Mb) and Chromosome 16 (0–4 Mb). Overall, the average recombination rate throughout the oil palm genome was estimated as 1.8 cM/Mb, ranging from 0.8 (Chromosome 5) to 2.9 cM/Mb (Chromosome 12) (Table 1).

### Improvement of genome coverage

**Figure 4:**
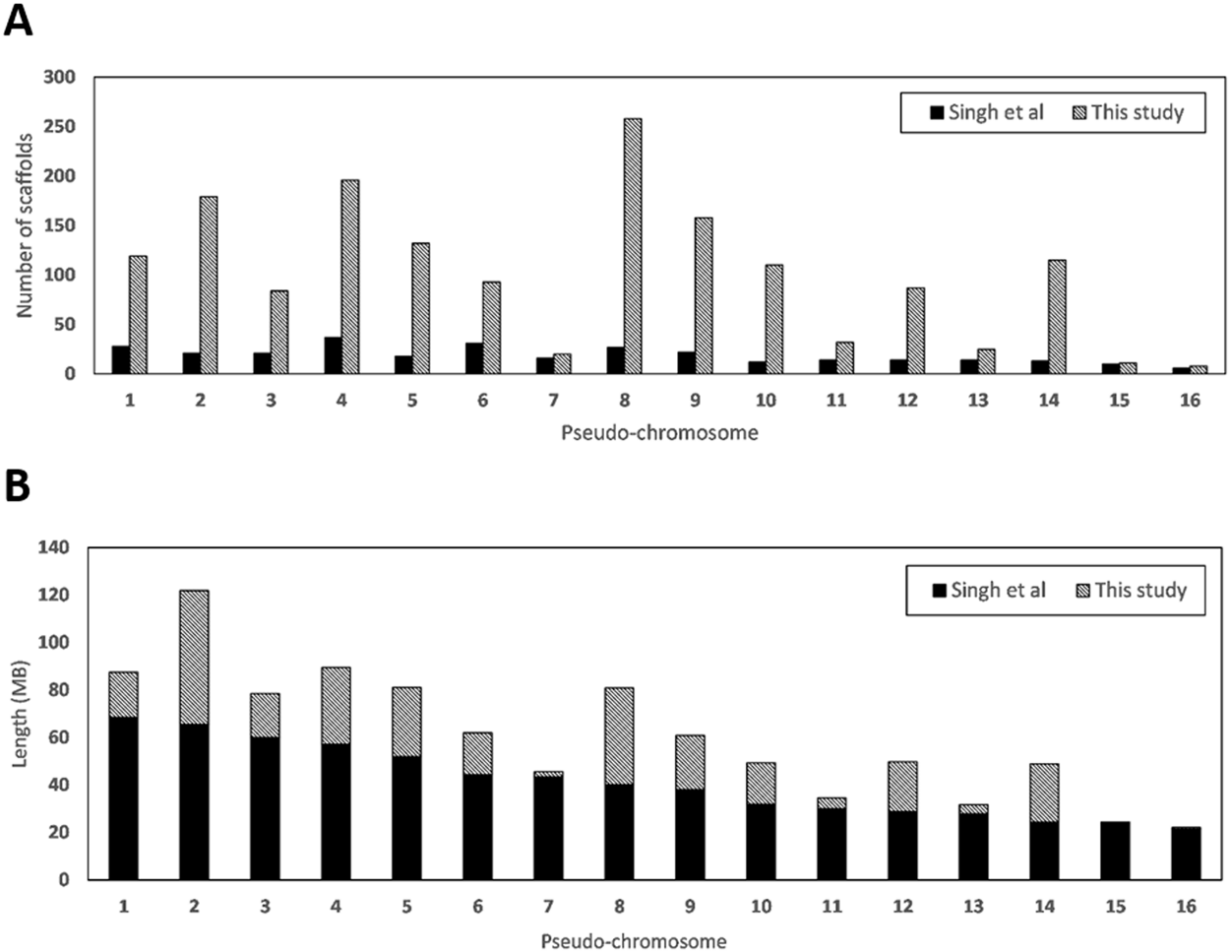
(**A**) Comparison of number of scaffolds from previous physical map^20^ and the improved physical map developed in this study. (**B**) Total length improvement (Mb) for each of the pseudo-chromosome after addition of new scaffolds to the published genome sequence in this study.

The average number of scaffolds contributing to genome sequences by Singh *et al*. (2013) are 19 scaffolds in each pseudo-chromosome. The ultrahigh-density linkage map identified a total of 311 Mb in scaffolds in addition to the existing genome assembly that could be added to the linkage groups. In total, 1,323 additional scaffolds were placed into pseudo-chromosome locations (Fig. 4A). Figure 4B depicted the total length for each pseudo-chromosome from published genome sequences. Each of the pseudo-chromosome was extended from this study with the most significant extension in Chromosome 2 at 56 Mb, while Chromosome 15 and 16 also have minimal extensions of less than 1 Mb, hence the improvements were not distinguishable in the plot (Fig. 4B). After scaffolds addition, the physical map was successfully extended from 658 Mb to 969 Mb, which is equivalent to a 47% improvement. Still, the improved physical map only covers 54% of the total genome based on the estimated genome size of oil palm of 1.8 Gb.

## Discussion

The published OP200K Infinium array was derived from 59 important and diverse origins^19^ and was used in this study. This helps to explain why only 13.9% (27,890) of the assayed SNPs were polymorphic and could be mapped in the advanced commercial Deli *dura* x AVROS *pisifera* cross. Still, the average mapping interval of 0.04 cM is the highest reported, compared to previous studies (1.26 cM^35^, 1.40 cM^10^ and 0.29 cM^36^). In fact, the improvement of the mapping interval was mainly due to higher recombination detected in this larger mapping population, which was double the size of the 153 *tenera* palms used by Bin Bai and colleagues. So far, only Lep-MAP and MSTmap^37^ programmes are able to map more than 10,000 SNP markers in under an hour. However, the MSTmap is limited to single families and phased data (including BC1, RIL, DH and Hap populations). The Lep-MAP does not have such limitations, so it was chosen to analyse the unphased cross-pollinating (CP) populations of oil palm. By using Lep-MAP3, the total length of the genomic SNP-based linkage map was significantly reduced from 2,938.2 cM obtained using Lep-MAP2^36^ to 1,151.7 cM. With 16 chromosomes, a combined linkage distance in the order of 1,600 cM would be expected. Lep-MAP3 is able to minimise inflated map length and ordering error when the number of markers is much higher than the mapping population size^31^. This is a critical improvement because incorrect map length can greatly affect subsequent recombination estimates.

For the genomic linkage map, each LG was named based to their published pseudo-chromosomes^20^ with 61.7% of linked SNP sequences found in the reference genome (Table 1). The oil palm consists of a diploid genome with 16 homologous chromosome pairs (2n = 2× = 32). In this map three LGs fragmented into two parts from LG 2, LG 5 and LG 7, respectively (Fig. 1). Similar LG fragmentation was also observed in the genic linkage map (Fig. 2A). In this case, LG 2.2 and LG 7.2 were absent because none of their SNP was genic, whereas removing non-genic SNPs on LG 11 caused fragmentation to two groups. More markers normally confer a longer map, reflecting better mapping/genome coverage, but also potentially reflecting a reduction in the data quality as more comprehensive use is made of the dataset. The phenomena were not observed in this study. The genic linkage map with only 2,314 SNPs (1,733 cM) was longer than that of the genomic linkage map with 27,890 SNPs (1,151.7 cM). Although linkage analysis is often problematic when the number of markers is high (approaching 10,000 markers). However, we found that both JoinMap 5 and Lep-MAP3 performed equally as reported^30,31^. The common SNP markers between the genomic and the genic linkage maps were in synteny (Supplementary Fig. 1), thus Lep-MAP3 is recommended.

Sugar and lipid-related biosynthesis pathways on the genic linkage map (Fig. 2) revealed genetic polymorphisms still present within the commercial *tenera* family which could be exploited in breeding program. Notably that there is still number of unannotated genes showing polymorphism within this population indicating novel genes might have important functions and to be discovered by further QTL mapping. The published QTLs for mesocarp oil content were located on Chromosome 5 and Chromosome 11 in Deli *dura* x AVROS *pisifera* population^18^. In the genic linkage map, the QTL region on Chromosome 5 was mainly populated by plant hormone signal transduction, glycerophospholipid and glycerolipid metabolism pathway genes. The plant hormone signal transduction pathways tagged by the most SNPs was closely related to plant floral development, growth and response to the environment^38^. As for the QTL region on Chromosome 11, included glycolysis and starch-and-sucrose metabolism pathways. However, double recombination should rarely occur in the genic linkage map with an average mapping interval of 0.75 cM, which is much lower than 10 cM threshold^39^ and the mapping function partly compensates for this. For future QTL mapping analyses for oil yield traits in oil palm the genic linkage map with 10-fold fewer markers can be used, while also assessing polymorphism associated with genes known to be involved in important pathways related to oil production and energy.

Recombination frequency between marker and causal gene can weaken predictability of a trait. One way to quantify this effect is through genome-wide LD decay estimation, but it is influenced by the pedigree of the material. This parameter differs across origins, or even populations^40,41^. In oil palm, longer LDs in the commercial populations have been observed, compared to wide germplasm materials^18,19,42^ and the evidence presented here suggests that maternal and paternal recombination patterns also vary, which potentially influences breeding strategies. A good reference genome is also a key enabler to identify and characterise causal genes, based on recombination and to compare differences in genetic order, which could be indicative of structural rearrangements when compared to the physical genome. We further evaluated the assembled reference genome by comparing this with the genomic linkage maps of *dura*, *pisifera* and *tenera*. In general, these three linkage maps were well integrated and consistent with the physical map, although differences in cM/Mb were consistently observed between the two parental maps. Telomeres are hotspots of recombination, with crossover events rarely happening at the centromeric region in the chromosomes of many species^43^. The lack of genetic distance observed in sections of Chromosome 6 and Chromosome 7 for the *dura* parent is likely to be an effect of non-informative markers for linkage in the *dura* parent and could well indicate identity-by-descent on the maternal side. Generally, a lack of polymorphism be due to identity-by-descent issues. More broadly, assignment of further scaffolds to the pseudo-chromosomes may allow more extensive genome coverage and could help to assign more genetic effects to specific chromosomes, rather than ‘group 17’ (unplaced) assignment, indicating a failure to be localised to a pseudo-chromosome (Fig. 4). The detected outliers and inversion of chromosome segments with respect to the reference genome also might reflect genuine rearrangements, mainly inversion and translocation or even duplications. Long-read sequencing approaches, such as Pacific Biosciences (PacBio)^44^ and Oxford Nanopore technologies^45^ along with optical mapping could be coupled with linkage mapping to confirm the extension of the genome from newly added scaffolds as well as to differentiate the genuine structural rearrangements from the errors^46^.

The evenness of recombination can also be assessed by recombination rates with respect to physical distance^47^. The average recombination rate for the commercial Deli dura x AVROS *pisifera* progeny was estimated at 1.8 cM/Mb (Table 1), which was comparable to 2.0 cM/Mb for a bi-parental recombination inbred lines (RILs) derived from a chickpea (*Cicer arietinum* L.) family^48^. On the other hand, the parameter was higher than that of fourteen maize full-sib families of DH lines with <1.0 cM/Mb^49^. Breeding design and genetic resources that influence the recombination landscape in the genome, usually receive little attention in mapping analysis of oil palm. For oil palm breeding, a variant of reciprocal recurrent selection (RRS) or family and individual selection (FIS) is deployed, producing thick-shelled dura maternal and shell-less *pisifera* paternal pools and hybrids with thin shells and more mesocarp^2^. The superior parents with good combining ability assessed through progeny testing are pollinated to produce thin-shelled *tenera* commercial productions, with each selection cycle taking up to 12–19 years. Thus, selection of mapping population mostly depends on the existing trials, which were originally established for conventional breeding programmes. Furthermore, recombination rates of Deli *dura* and AVROS *pisifera* were also different in this study, further supporting their distinct selection histories in the past. The Deli *dura* are (supposedly) direct descendants of the four palms planted in 1848 in the Bogor Botanical Garden, Indonesia, whereas the AVROS largely originated from the well-known Djongo palms in Congo^2^. We found that the highest rates of recombination in the *tenera* were inherited from the AVROS *pisifera*, but not on all chromosomes (Fig. 3). This may partly be an effect of uninformative meiotic events due to identity-by-descent in the *dura* which appears greater here, but the consistent trend on many chromosomes of higher recombination rates in the *pisifera* parent than the *dura* parent suggests a real effect. The results agree with the presence of limited polymorphism in particular chromosomes, partly reflecting the narrow genetic base of Deli *dura* due to generations of self-pollination or sib-mating originated from the four ancestors^21^. This findings pave the way for beginning to understand the effects of selection pressure in both parent pools and how these might be combined to achieve additional heterosis in oil palm.

This study provides a good foundation to improve the genome coverage in the oil palm genome assembly, to unlock the full potential of genome-enabled biology for oil palm. By using a high-density SNP array and a population of 295 palms, we have been able to assign a further 311 Mb of scaffolds to the pseudo-chromosomes of Singh *et al*.^20^. However, this additional 311 Mb has been assigned according to the linkage order in the Deli *dura* x AVROS *pisifera* cross (278 x TT41/4) used in this study, which may differ from the actual scaffold order (and, ultimately, the genome sequence order) present in the original *pisifera* used to generate the genome sequence. An understanding of these differences will be critical to fully understand GxE and GxG interactions, as well to begin to unravel the underlying basis for some trait QTL having strong effects in one origin, but none in others. The development of a genic-based map also allows breeders to search for QTLs for important oil yield traits with a smaller subset of candidate genes from known pathways. More importantly, the recombination landscape across parents potentially permits understanding of selection pressure in the genome of oil palm and where further improvement may be possible. Appropriate experimental designs and genetic resources are equally important as marker interval and population size to further improve the map quality and QTL searches, both through biparental and population approaches.

## Supplementary information

Supplementary Info
