# Supplementary material for: Linkage-based genome assembly improvement of oil palm (Elaeis guineensis)
Source: Sci Rep. 2019 Apr 29;9:6619. doi: 10.1038/s41598-019-42989-y (PMC6488618; doi:10.1038/s41598-019-42989-y)

**Linkage-based genome assembly improvement of oil palm *(Elaeis guineensis)***

Ai-Ling Ong ^1,2*^, Chee-Keng Teh ^1,2^, Qi-Bin Kwong ^1^, Praveena Tangaya^1^, David Ross Appleton ^1^, Festo Massawe ^2^ , Sean Mayes ^3^

1. Biotechnology & Breeding Department, Sime Darby Plantation R&D Centre, Selangor, Malaysia
2. School of Biosciences, The University of Nottingham Malaysia Campus, Semenyih, Malaysia
3. School of Biosciences, University of Nottingham, Nottingham, UK.

***** Correspondence: [ong.ailing.sdtc@simedarbyplantation.com](file:///\\172.16.147.23\Jupiter\Bioinformatics\OAL\Pop%20Linkage%20Map\Mapping\Write-up\SciRep\ong.ailing.sdtc@simedarbyplantation.com); Tel.: +603-89422641

**Supplementary Table 1**

Summary of lower density Deli *dura* x AVROS *pisifera* genic SNP-based linkage map annotated from transcriptomic databases.

| **Linkage group** | **Linkage Length (cM)** | **No. of selected genic SNPs** |
| --- | --- | --- |
| 1 | 162.33 | 215 |
| 2 | 235.1 | 194 |
| 3 | 156.63 | 260 |
| 4 | 146.03 | 134 |
| 5 | 68.02 | 84 |
| 5.2 | 13.61 | 18 |
| 6 | 105.806 | 117 |
| 7 | 103.90 | 120 |
| 8 | 119.34 | 178 |
| 9 | 94.51 | 120 |
| 10 | 140.90 | 310 |
| 11 | 47.15 | 63 |
| 11.2 | 29.92 | 35 |
| 12 | 69.62 | 123 |
| 13 | 49.76 | 41 |
| 14 | 72.58 | 165 |
| 15 | 72.43 | 88 |
| 16 | 46.40 | 49 |
| **Total** | **1733.01** | **2314** |
| **Mean** | **96.28** |  |

**Supplementary Figure 1**

The common SNP markers between the genomic and the genic maps which were constructed using Lep-MAP 3 and JoinMap 5, respectively. High concordance of marker ordering were observed for lower density and ultra-dense genetic maps. Chromosomes 16, 14 and 8 are shown as examples.


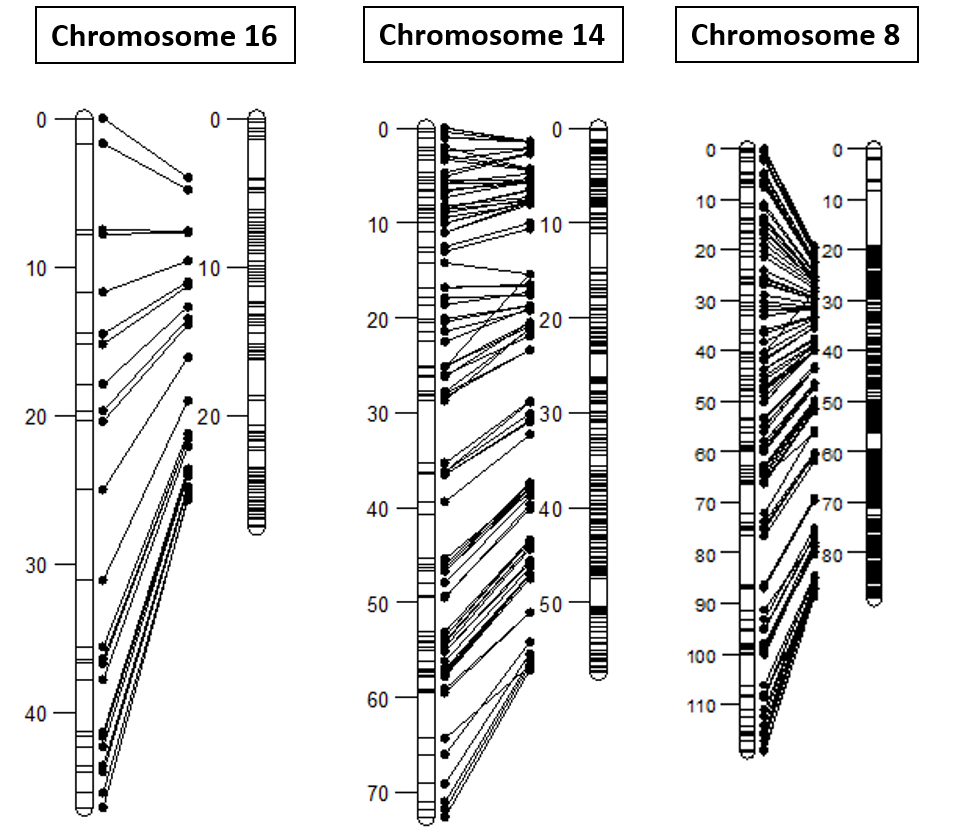

Supplement: Supplementary file 1 — Supplementary Info [file 41598_2019_42989_MOESM1_ESM.docx]
